# The Association Between Precuneus Function and Residual Dizziness in Patients With Benign Paroxysmal Positional Vertigo

**DOI:** 10.3389/fneur.2022.828642

**Published:** 2022-04-12

**Authors:** Wei Fu, Ya Bai, Feng He, Dong Wei, Yuanyuan Wang, Ying Shi, Xinyue An, Junliang Han, Xiaoming Wang

**Affiliations:** ^1^Department of Geriatrics, Xijing Hospital, Fourth Military Medical University, Xi'an, China; ^2^Department of Neurology, Xijing Hospital, Fourth Military Medical University, Xi'an, China

**Keywords:** benign paroxysmal positional vertigo, residual symptoms, precuneus, brain function, questionnaires

## Abstract

**Objectives:**

The purpose of this study was to apply the amplitude of the low-frequency fluctuation (ALFF) method to investigate the spontaneous brain activity alterations in patients with residual dizziness (RD) after successful canalith repositioning manoeuvre for benign paroxysmal positional vertigo (BPPV).

**Methods:**

All BPPV patients underwent visual vertigo analog scale (VVAS) evaluations and functional magnetic resonance imaging (fMRI). The ALFF method was used to assess the spontaneous brain activity. Screening of brain regions with significant changes in ALFF values was based on analysis of the whole brain. We further analyze the relationship between ALFF values of the altered regions and VVAS scores in BPPV patients with RD.

**Results:**

Fifteen BPPV patients with RD and fifteen without RD were recruited in this study. In contrast to without RD, RD patients exhibited increased scores in VVAS tests (*p* < 0.001) and RD patients also showed significant ALFF decrease in the bilateral precuneus (left: 251 voxels; *x* = −10, *y* = −69, *z* = 51; peak t-value = −3.25; right: 170 voxels; *x* = 4, *y* = −59, *z* = 42; peak t-value = −3.43). Correlation analysis revealed that the mean ALFF z-values in the left precuneus displayed significant negative correlations with the VVAS scores(*r* = −0.44, *p* = 0.01).

**Conclusions:**

This study shows that RD is associated with left precuneus function as revealed by fMRI. It might provide useful information for explaining neural mechanisms in BPPV patients with RD.

## Introduction

Benign paroxysmal positional vertigo (BPPV) is the most common cause of peripheral vestibular vertigo (1). Treatment of BPPV relies on canalith repositioning maneuver (CRM) (2). Despite CRM being a successful treatment of BPPV, some patients still report a non- specific sensation of unsteadiness, lightheadedness, disorientation, fogginess, or drowsiness named residual dizziness (RD) (3). Studies reported that incidence of RD ranges from 29.6 to 61%, and the duration of RD can range from a few weeks to several months (4–6). Thus, additional evaluation and management are important for RD patients. However, the mechanism of RD is still unknown. Some studies considered that delayed recovery might be due to the longer time needed for vestibular information adaptation in the central nervous system (7).

Resting-state functional magnetic resonance imaging (rs-fMRI) is a reliable and non-invasive technique that has a good signal-to-noise ratio, requires minimal subject compliance, and is well suited for exploring the brain and central nervous system (8). Amplitude of low-frequency fluctuation (ALFF) is an advanced approach for analysis of rs-fMRI data that uses voxel-based analysis and directly reflects the intensity of spontaneous neuronal activity in the baseline state (9).

A recent rs-fMRI study found that cerebellum structural and functional changes in patients with BPPV, which may reflect the central adaptation and plasticity after BPPV treatment (10). Apart from the cerebellum, the processing of vestibular information also involves the precuneus. Some studies show that electrical stimulation of the precuneus may induce the symptom of dizziness (11, 12). However, the effects of precuneus function on RD are yet to be investigated. In order to explore the precuneus function of RD patients. We aim to detect the brain regions with significant changes in ALFF values based on an analysis of the whole brain. And we further reveal the correlation between precuneus function and RD in patients with BPPV.

## Materials and Methods

### Patients

Between June 2021 and December 2021, Fifteen BPPV patients with RD and fifteen without RD were recruited at the neurotology unit of Xijing Hospital. All patients had undergone successful CRM treatment and routine nervous system examination. The diagnosis of BPPV was performed using the diagnostic criteria established by the Bárány Society in 2015 (13). The exclusion criteria for this study were: (1) multicanal or superior canal BPPV; (2) CRM treatment failure; (3) coexisting other vestibular disorders, such as vestibular neuritis, vestibular migraine; (4) central nervous system disorders; (5) contraindications for MRI examination. All the patients were right-handed. Informed consent was obtained from all patients before their inclusion in the study. The study was approved by the Institutional Review Board of Xijing Hospital, Fourth Military Medical University.

### Canalith Repositioning Maneuvers

Patients with posterior semicircular canal BPPV were treated by Epley's maneuver (2). Patients with geotropic lateral canal BPPV were treated by barbecue rotation maneuver (14). Patients with apogeotropic lateral canal BPPV were treated by Gufoni maneuver (15). A Dix–Hallpike or Roll test maneuver was repeated about 60 min after the treatment and CRP was defined as “successful” if positional nystagmus was no longer detectable and the patient did not complain of vertigo. Otherwise, the first CRM was defined as “ineffective” and repeated up to three times, checking the treatment result following each CRM. The patients of successful CRM treatment had undergone 1 week follow-up evaluation *via* interviews at the outpatient clinic after first visit. During the follow-up, patients again underwent diagnostic positional tests. In case of negative test results (absence of positional vertigo and undetectable positional nystagmus), the identification of RD was subsequently performed. On the basis of successful CRM treatment, we performed a structured interview procedure: (1) the first question aimed to identify the persistent dizziness symptoms. We asked “Do you still feel unsteadiness, lightheadedness, disorientation, fogginess, or drowsiness during the past days?”. In case the answer was “YES”, we further asked the next question. (2) the second question aimed to verify the onset of RD. We asked “is there a significant relief for symptoms after CRM?”. If the answer was “No”, the patient was considered to have RD. Eligible patients underwent RD evaluations and fMRI scans.

### Clinical Features and RD Evaluations

The clinical characteristics of the patients were recorded, including age, sex, duration of vertigo before CRM, involved canal, and affected side. In addition, all patients need to complete the Visual vertigo analog scale (VVAS) questionnaire (16). The VVAS used a straight line of 10 cm in length. The numbers between 0 and 10 were marked at equal intervals along the line. The patients were asked to report their self-perceived dizziness on a VVAS describing the severity of dizziness in everyday life on a continuum from 0 (none) to 10 (extremely severe).

### MRI Acquisition

All brain MR imaging was acquired using a 3-Tesla MR scanner (GE Discovery MR750 3.0T scanner). High-resolution T1-weighted 3D anatomical data were acquired using the 3D magnetization-prepared rapid gradient echo (3D MPRAGE) sequence (repetition time: 2530 ms; echo time: 3.5 ms; flip angle: 7°; field of view: 256 × 256 mm; matrix: 256 × 256; slice thickness: 1 mm; section gap: 0 mm; number of slices: 196). The image resolution was 1 × 1 × 1 mm. The echo planar imaging (EPI) sequence (repetition time: 2000 ms; echo time: 30 ms; flip angle: 90°; field of view: 240 × 240 mm; matrix: 64 × 64; slice thickness: 4 mm; section gap: 0.6 mm; number of slices: 45) effectively covered the entire brain. A custom-built head coil cushion and earplugs were used to minimize head motion and dampen scanner noise. During data acquisition, subjects were asked to remain alert with eyes closed and keep their head still.

### MRI Data Preprocessing and ALFF Analysis

The functional MRI (fMRI) data were analyzed using the Data Processing Assistant for Resting-State fMRI DPARSF toolbox based on the Matrix Laboratory platform (MATLAB R2013a; MathWorks, Cambridge, MA) (17). The procedures of MRI data was processed as follows: (1) convert image data from DICOM format to NIFTI format; (2) deletion of the first 10 time points of the functional images: the first 10 time points were deleted to address the possible instability of the initial MRI signal and the need for participants to adapt to the scanning environment; (3) slice time correction: the 23rd slice was chosen as the reference slice; (4) realignment: slight head motion of the subject between the time points during the scan was corrected. To ensure the accuracy of the position information, subjects who had more than 2 mm head translation in x, y, or z and 2° head rotation were removed; (5) regression: a linear regression model was used to remove the interference signal in the blood oxygen level dependent (BOLD) signal; (6) spatial normalization: the remaining functional images were spatially normalized using an echo-planar imaging standard template from the Montreal Neurological Institute (MNI) and resampled at a resolution of 3 × 3 × 3 mm; (7) spatial smoothing: smoothing was performed with a Gaussian kernel of 4 × 4 × 4 mm to reduce registration errors and increase the normality of the data; (8) ALFF calculation: ALFF was computed based on Fast Fourier transform (FFT) and the time series of each voxel was transformed to frequency domain without band-pass filtering. The square root was first calculated at each frequency of the power spectrum, and then the mean square root was obtained across 0.01–0.08 Hz band for each voxel. Each individual's ALFF value was then transformed to a Z-score to allow further comparison between groups.

### Statistical Analysis

All continuous variables are reported with mean values and standard deviation and categorical variables with absolute and relative frequencies. Statistical analysis included Student's *t*-test for continuous variables, the chi-square test and Fisher's exact test to compare proportions. Statistical analysis were done by using SPSS software (version19, SPSS Inc., Chicago, IL, USA) with a statistical significance at *p* < 0.05.

The two-sample *t*-test was used to compare the ALFF values in each voxel of the two groups. Multiple comparisons at cluster level using non-parametric permutation test can easily control the false positive rate existing in statistics and 5,000 permutation times and the prominent cluster size > 30 voxels were performed in this work (two-tailed, threshold level: *p* < 0.05) (18, 19). The relationship between the mean ALFF z-values in significantly altered regions and VVAS scores in the RD and without RD groups was calculated using Pearson correlation by using SPSS software (version19, SPSS Inc., Chicago, IL, USA).

## Results

### Clinical Characteristics of BPPV Patients With and Without RD

Clinical characteristics of patients with BPPV patients with and without RD are described in Table 1. There were no significant differences between the RD and without RD groups in age, sex, duration of vertigo, involved canal, and affected side (*p* > 0.05). There was a significant difference in VVAS scores between the RD and without RD groups (*p* < 0.001).

**Table 1:** Demographic and clinical characteristics of patients with and without RD.

**Features**	**RD (*n* = 15)**	**Without RD (*n* = 15)**	***p*-value**
Age (years, mean ± SD)	64.40 ± 13.27	56.53 ± 8.06	0.06
Gender (%)			
Female Male	14 (93.33) 1 (6.67)	9 (60.00) 6 (40.00)	0.08
Involved canal (%)			
Posterior Horizontal	12 (80.00) 3 (20.00)	13 (86.66) 2 (13.34)	1.00
Affected side (%)			
Right Left	6 (52.32) 9 (47.68)	9 (56.75) 6 (43.25)	0.273
Duration of vertigo (days, mean ± SD)	14.40 ± 8.39	11.46 ± 6.71	0.30
VVAS score (mean ± SD)	6.46 ± 1.80	2.33 ± 1.44	<0.001

### ALFF Differences Between RD and Without RD

**Figure 1:**
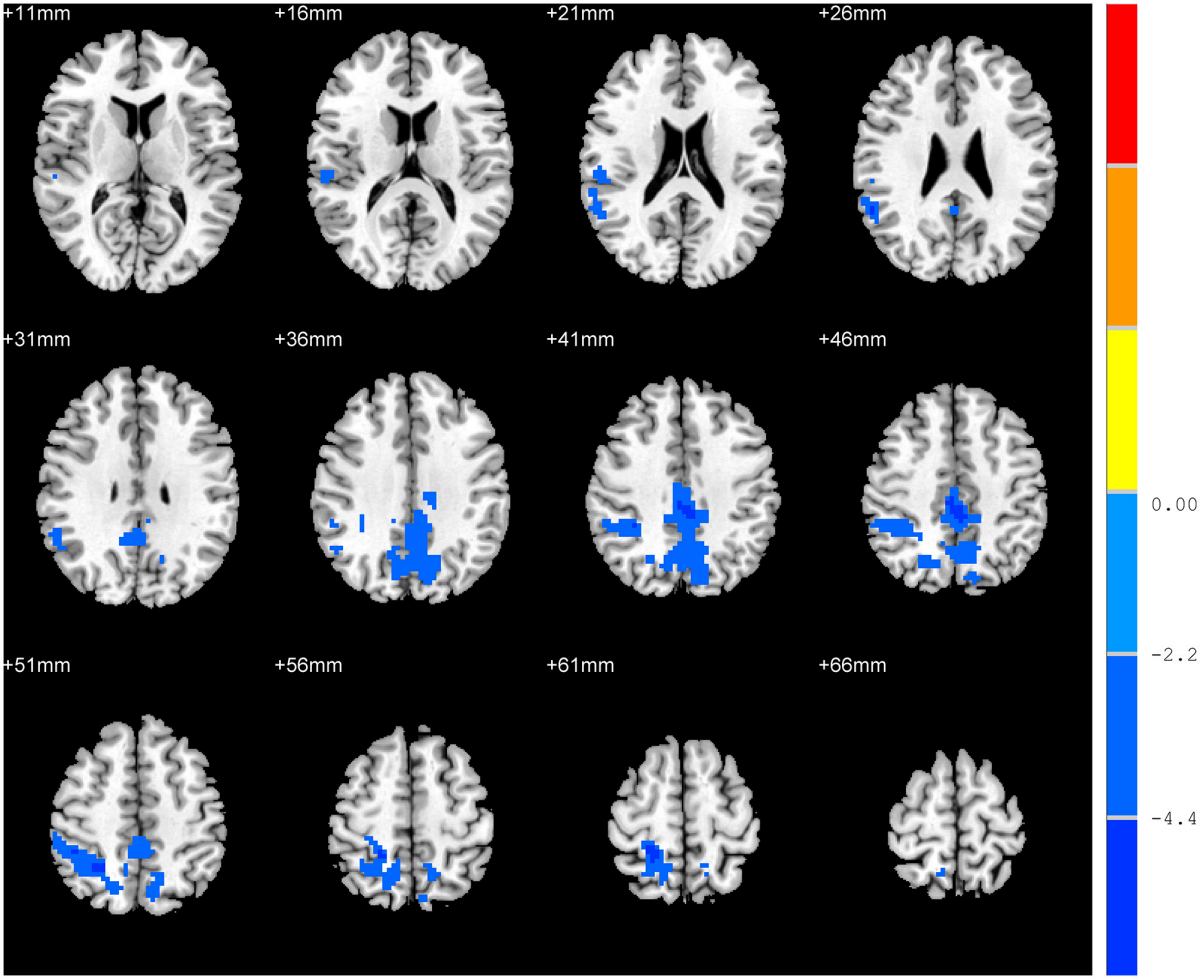
Differences in the amplitude of low-frequency fluctuation between residual dizziness group and without residual dizziness group.

In contrast to without RD, the RD patients showed significant ALFF decrease in the bilateral precuneus, right inferior parietal lobule, bilateral middle cingulate gyrus, right superior parietal lobule, right supramarginal gyrus, and right angular gyrus (*p* < 0.05, Table 2, Figure 1).

**Table 2:** Brain regions showing significant differences in ALFF values between the RD and without RD groups.

**Brain region (AAL)**	**Number of voxels**	**MNI coordinates (x, y, z)**	**Peak t value**
Precuneus_L	251	−10, −69, 51	−3.25
Precuneus_R	170	4, −59, 42	−3.43
Parietal_Inf_R	157	37, −45, 49	−3.64
Cingulate_Mid_L	163	−2, −34, 47	−4.78
Parietal_Sup_R	97	17, −66, 52	−3.41
Cingulate_Mid_R	69	2, −47, 36	−3.48
Supra Marginal_R	74	60, −48, 26	−4.65
Angular_R	35	55, −52, 25	−4.78

### Correlation Analysis

**Figure 2:**
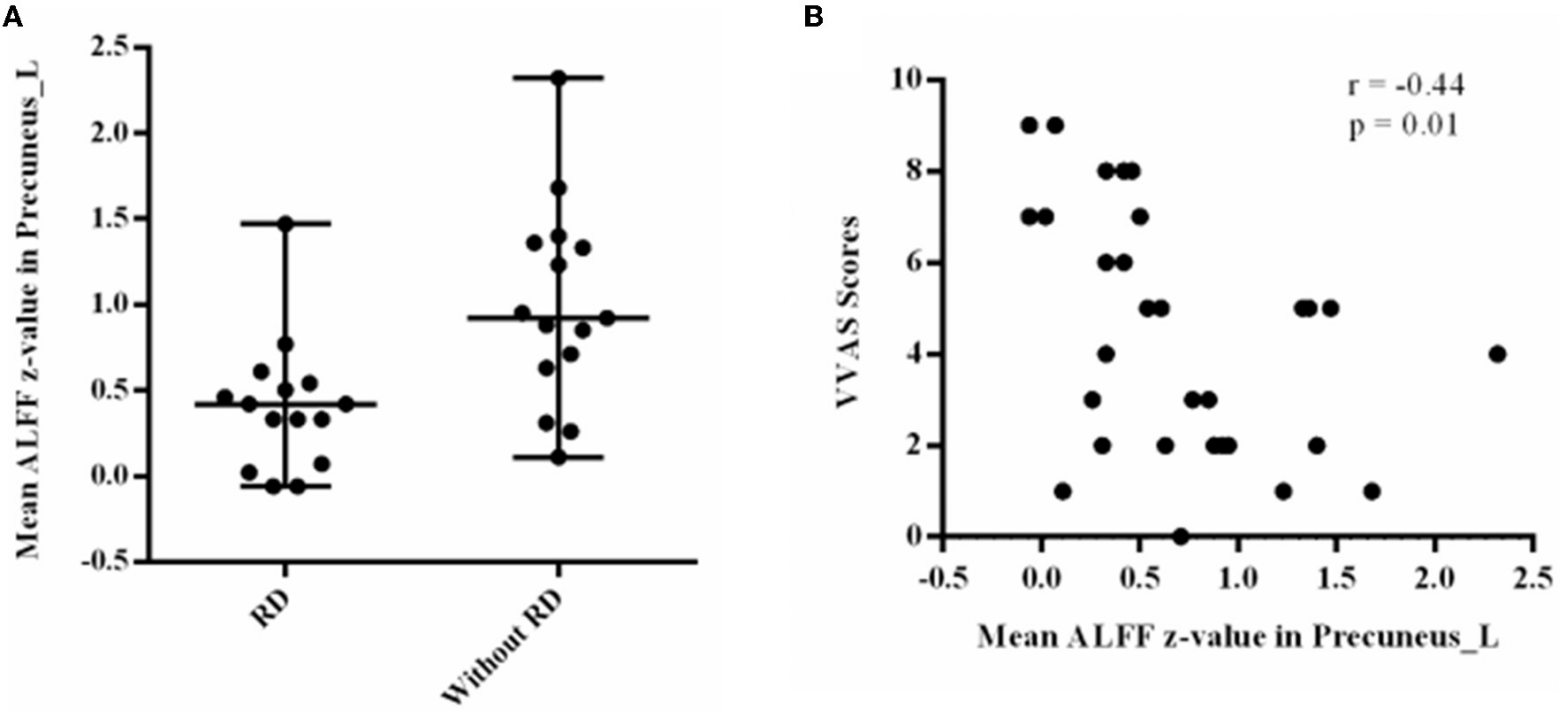
**(A)** Residual dizziness patients showed significant amplitude of low-frequency fluctuation decrease in the left precuneus. **(B)** Correlation analysis revealed that the mean amplitude of low-frequency fluctuation z-values in the left precuneus displayed significant negative correlations with the visual vertigo analog scale.

Correlation analysis revealed that the mean ALFF z-values in the left precuneus displayed significant negative correlations with the VVAS scores (*r* = −0.44, *p* = 0.01, Figure 2). There was no significant correlation between the mean ALFF z-values in other brain regions and VVAS scores (*p* > 0.05).

## Discussion

In our study, we compared the VVAS scores and resting-state ALFF in the brains of patients with RD and without RD. RD patients exhibited increased scores in VVAS tests and significantly reduced ALFF in the bilateral precuneus, right inferior parietal lobule, bilateral middle cingulate gyrus, right superior parietal lobule, right supramarginal gyrus, and right angular gyrus. Additionally, the mean ALFF z-values in the left precuneus displayed negative correlations with the VVAS score.

Previous study have shown that VVAS is increased in RD patients (20). Our study is consistent with previous study. In our study, the mean values of VVAS scores are 6.46 in RD patients. It demonstrated that RD patients still have moderate to severe dizziness after CRM treatment. Thus, exploring the mechanism of RD is important for BPPV patients.

The ALFF method has been applied to evaluate the neural mechanisms of central disease including schizophrenia (21), epilepsy (22), mild cognitive impairment (23), and migraine (24). Besides, many studies have suggested that the ALFF of fMRI signals are closely related to the spontaneous neuronal activities such as visual cortex (25), auditory cortex (26), hippocampus (27), cingulate cortex, and postcentral gyrus (28). We applied the ALFF method to investigate the spontaneous brain activity alterations in patients with RD and found significant ALFF decrease in the bilateral precuneus. The precuneus, located in the posteromedial parietal lobe, is considered to play a central role in a range of highly integrated tasks, including cognitive tasks (29, 30) and perception of self-motion (31). Some studies also showing that electrical stimulation of the precuneus may induce the symptom of dizziness (11, 12). Thus, the precuneus is a key brain region in processing vestibular information. Besides, a previous PET study confirmed the concept of inhibitory visual-vestibular interaction in the cortex and regional cerebral blood flow increased in inferior parietal lobule by caloric vestibular stimulation and visual fixation (32). Unlike previous studies, we applied resting-state fMRI to investigate the spontaneous brain activity alterations in patients with RD and found significant ALFF decrease in vestibular-related areas. There are some explanations for this difference: firstly, there are differences in research methods. Previous studies used visual or vestibular stimulation. However, our studies did not give any stimulus. Secondly, a mechanism of RD is that the brain delays recovery for central adaptation after particle repositioning. Our present results will add to neuroimaging evidence to display delayed recovery of central adaptation in the brain. A recent rs-fMRI study found cerebellum structural and functional changes in BPPV patients with RD, which may reflect the central adaptation and plasticity (10). However, the study focused on the cerebellum but not on the precuneus. Therefore, our study provide the direct evidence that the precuneus is linked to RD. Moreover, we further found that VVAS scores of RD patients displayed negative correlations with the mean ALFF z-values in the left precuneus. VVAS mainly assess the dizziness in dynamic visual environments. A previous study also reported that precuneus has been associated with the visuospatial circuit in processing visual information (33). Therefore, the precuneus is associated with the multisensory interactions between the vestibular and visual area. A previous study also found that functional connectivity altered between the associative visual cortex and precuneus in dizziness patients (34). Previous report and our study indicated the abnormal integration of visual information and vestibular information. Our results add to the importance of the precuneus in multisensory interactions.

Apart from the precuneus, we also found that the spontaneous brain activity of RD patients was impaired in the right inferior parietal lobule, bilateral middle cingulate gyrus, right superior parietal lobule, right supramarginal gyrus, and right angular gyrus. According to current research, vestibular processing involved multiple multisensory vestibular cortex areas. Multiple sensory inputs converge at all levels of the central vestibular system from the vestibular nuclei to the temporoparietal cortex (35). Functional imaging studies in humans using caloric and galvanic vestibular stimulation revealed that the inferior parietal lobule, anterior cingulum, anterior insula, and hippocampus all belong to the multisensory vestibular cortical network (36). This network is found primarily in the temporoinsular and temporoparietal cortex and has been delineated in both human hemispheres (37). A meta-analysis and conjunction analysis confirmed that the parietal operculum as the core region for vestibular processing was directly connected with temporoparietal regions, premotor cortex, and the midcingulate gyrus (38). Another meta-analysis also tried to statistically analyze the localization of human vestibular cortex areas for different types of stimulations revealed main regions in the insula, retroinsular cortex, frontoparietal operculum, and cingulate cortex (39). Furthermore, in humans, vestibular inputs also elicit activity in the angular gyrus, supramarginal gyrus of the inferior parietal lobule, and the superior parietal lobule (40). In total, the multisensory vestibular cortex area is a complex function network. Future research is needed to further uncover the interrelationships between these functional networks.

## Conclusions

In conclusion, this study shows that RD are associated with left precuneus function as revealed by fMRI. It might provide useful information for explaining neural mechanisms in BPPV patients with RD.

## Data Availability Statement

The original contributions presented in the study are included in the article/supplementary material, further inquiries can be directed to the corresponding author/s.

## Ethics Statement

The studies involving human participants were reviewed and approved by Institutional Review Board of Xijing Hospital, Fourth Military Medical University. The patients/participants provided their written informed consent to participate in this study.

## Author Contributions

WF designed the experiment, analyzed the data, and wrote the article. FH and YB recruited patients. DW, YW, YS, and XA collected data. JH and XW guided the study. All authors contributed to the article and approved the submitted version.

## Funding

This study was supported by the National Key R&D Program of China (No. 2018YFC2000300) and the Key R&D Program of Shaanxi Province (No. 2022SF-283).

## Conflict of Interest

The authors declare that the research was conducted in the absence of any commercial or financial relationships that could be construed as a potential conflict of interest.

## Publisher's Note

All claims expressed in this article are solely those of the authors and do not necessarily represent those of their affiliated organizations, or those of the publisher, the editors and the reviewers. Any product that may be evaluated in this article, or claim that may be made by its manufacturer, is not guaranteed or endorsed by the publisher.
